# Parental Control, Nurturance, Self-Efficacy, and Screen Viewing among 5- to 6-Year-Old Children: A Cross-Sectional Mediation Analysis To Inform Potential Behavior Change Strategies

**DOI:** 10.1089/chi.2014.0110

**Published:** 2015-04-01

**Authors:** Russell Jago, Lesley Wood, Jesmond Zahra, Janice L. Thompson, Simon J. Sebire

**Affiliations:** ^1^ Centre for Exercise, Nutrition and Health Sciences, School for Policy Studies, University of Bristol, Bristol, United Kingdom.; ^2^ School of Sport, Exercise and Rehabilitation Sciences, University of Birmingham, Birmingham, United Kingdom.

## Abstract

***Background:*** Children's screen viewing (SV) is associated with higher levels of childhood obesity. Many children exceed the American Academy of Pediatrics guideline of 2 hours of television (TV) per day. There is limited information about how parenting styles and parental self-efficacy to limit child screen time are associated with children's SV. This study examined whether parenting styles were associated with the SV of young children and whether any effects were mediated by parental self-efficacy to limit screen time.

***Methods:*** Data were from a cross-sectional survey conducted in 2013. Child and parent SV were reported by a parent, who also provided information about their parenting practices and self-efficacy to restrict SV. A four-step regression method examined whether parenting styles were associated with the SV of young children. Mediation by parental self-efficacy to limit screen time was examined using indirect effects.

***Results:*** On a weekday, 90% of children watched TV for <2 hours per day, decreasing to 55% for boys and 58% for girls at weekends. At the weekend, 75% of children used a personal computer at home, compared with 61% during the week. Self-reported parental control, but not nurturance, was associated with children's TV viewing. Parental self-efficacy to limit screen time was independently associated with child weekday TV viewing and mediated associations between parental control and SV.

***Conclusions:*** Parental control was associated with lower levels of SV among 5- to 6-year-old children. This association was partially mediated by parental self-efficacy to limit screen time. The development of strategies to increase parental self-efficacy to limit screen-time may be useful.

## Introduction

Higher levels of screen viewing (SV) are associated with higher levels of obesity among children.^1,2^ Several studies have reported that many children exceed the American Academy of Pediatrics (AAP) guideline to limit noneducational screen time for children older than 2 years of age to a maximum of 2 hours per day.^3,4^ Given that SV tracks from childhood into adulthood,^5^ ensuring that children moderate their screen time is likely to help prevent future obesity.

Identification of the variables associated with a behavior is a critical first step in designing new interventions.^6,7^ Parents play a pivotal role in children's behavior, and it seems likely that parents influence children's SV. Parenting styles set the emotional context of parent-child interactions.^8–10^ Maccoby and Martin^11^ defined four parenting styles. Authoritative parenting (high nurturance, high control) is associated with positive child outcomes, including higher academic performance^12,13^ and fruit and vegetable intake.^14,15^ The remaining three styles are: authoritarian (low nurturance, high control); indulgent (high nurturance, low control); and uninvolved (low nurturance, low control). Parenting styles are based on the extent to which the parent adopts a nurturing and controlling communication style.

Several systematic reviews have examined whether parenting practices are related to child SV.^16–18^ These reviews suggest that a range of different parenting measures have been used, but the reliability and validity of those measures and inconsistencies in study design mean that more work on parental factors associated with child SV is needed.^16–18^ Self-efficacy to manage screen time has been associated with lower levels of television (TV) viewing among children,^19^ and there is some evidence that parental self-efficacy to manage SV is associated with lower SV among UK and Australian preschool-aged children.^20,21^ There is little information about whether parental self-efficacy to limit child screen time is associated with SV among children at the start of primary school, a key period for the development of obesity and SV behaviors.^1^

Sleddens has proposed a conceptual model of how parents influence children's diet and physical activity.^22^ Application of the model to SV and self-efficacy would suggest that any link between parental control/nurturance and child SV is likely to be partially mediated by parental self-efficacy to limit SV. The aims of this study were therefore to examine whether (1) parental control or parental nurturance were associated with SV in young children, (2) parent self-efficacy to limit SV was associated with SV in young children, and (3) any association between either parental control or parental nurturance and SV in young children was mediated by self-efficacy to limit screen time.

## Methods

Data are from the B-ProAct1v cross-sectional study.^23,24^ Details of the study design have been reported elsewhere.^23,24^ Briefly, between January 2012 and July 2013, 250 primary schools were invited to participate in the study. Data were collected in 57 schools that responded and for which data collection could be scheduled.^24^ The study was approved by a University of Bristol (Bristol, UK) ethics committee, and written informed consent was obtained for all participants.

A parent (mother or father) completed a questionnaire that included questions about their own SV behavior and that of their child, as well as parenting constructs. Parents reported their own and their child's SV, with separate questions relating to TV viewing and computer/laptop use. For each SV device, the parent was asked to report the time he or she and their child spent using it during a (1) normal weekday and (2) normal weekend day, with the following response options: none; 1–30 minutes; 31 minutes–1 hour; 1–2 hours; 2–3 hours; 3–4 hours; or >4 hours. The assessment of TV viewing using parental response to a single question has been shown to correlate moderately (*r*=0.60) with 10 days' of TV diaries among young children.^25^

Parental control and nurturance were measured using the Parental Dimension Index.^26^ The nurturance subscale consists of six items, including “I encourage my child to talk about his or her troubles.” Responses were measured on a Likert scale with a range of “Not at all like me” (scored as 1) to “Exactly like me” (scored as 6) and a total score of 6–36. The control subscale comprises five pairs of opposing statements with respondents being asked to choose the statement that they agree with most closely. The scale has a range of 0 (low control) to 5 (high control).

Parents' self-efficacy to reduce the child's SV behavior was measured using three questions that were based on Bandura's recommendations.^27^ The questions were: (1) “How much can you do to control the time your child spends SV (*e.g*., watching TV, digital video discs [DVDs], or playing video games)?”; (2) “How much can you do to help your children have alternatives to screen-viewing?”; and (3) “How much could you do to reduce the time your child spends screen-viewing?” Responses ranged from “Nothing” (scored 1) to “A great deal” (scored 5).

Parents self-reported height and weight, and BMI (kg/m^2^) was calculated. Home postcode was used to derive the index of multiple deprivation (IMD), with a higher score indicating greater deprivation.

### Data Preparation

Based on the AAP guidance, children's TV viewing behavior was collapsed into two categories (<2 hours per day and ≥2 hours per day).^3^ Children's screen use for other devices was collapsed into two categories of “no use” and “some use.”

### Statistical Analysis

Descriptive statistics were calculated. Cronbach's alpha was used to investigate the internal consistency of the parenting variables. The parental nurturance scale was internally consistent (alpha=0.864). The parental control measure had low internal consistency (alpha=0.302), and exploratory factor analysis indicated that the items did not load onto a single factor. However, given the low number of items, we retained the summary variable in all analyses. A factor analysis indicated that all three items designed to measure parental efficacy to limit SV loaded onto the same extracted latent variable and had good internal consistency (alpha=0.879).

Differences between parental genders were examined using chi-square and Student's *t*-tests. Differences in key variables between parents and children who did and did not provide sufficient information to be included in this analysis were also examined.

Preliminary correlation tests were used to examine whether mediation could have occurred^28^ and whether there was an association between the control and nurturance variables. Parental control and nurturance were both correlated with parental self-efficacy to influence SV (*r*=0.16; *p*<0.001 and *r*=0.27; *p*<0.001, respectively). There was, however, no evidence of an association between parental control and nurturance (*r*=0.027), and as such, there was no basis for further examination of the link between these two variables in the models.

**Figure 1.**
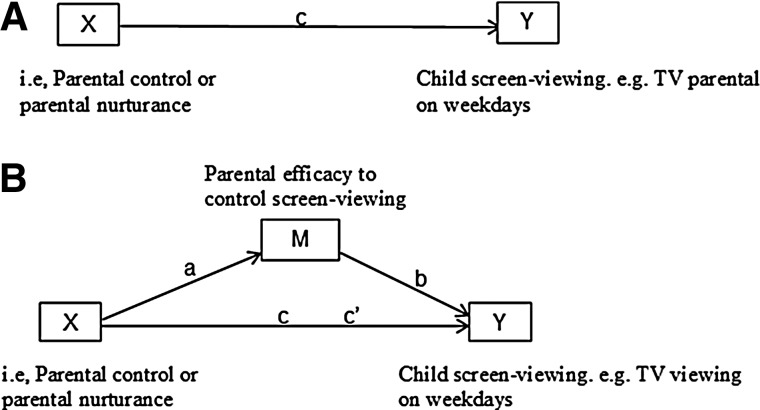
**(A) Direct relationship between parental control/nurturance and child screen viewing behavior. (B) Relationship between parental control/nurturance and child screen viewing behavior mediated by parental self-efficacy. TV, television.**

The hypothesis that any association between parental control or nurturance and child SV behavior was mediated by parental efficacy to influence SV (*aims 1 and 3*), was examined using the four-step regression approach recommended by Cerin and MacKinnon,^28,29^ which is summarized in Figure 1A and B. The four-step method involves testing a direct path between the exposure and the outcome and then estimating by how much this association is reduced by the inclusion of the potential mediator. We tested two exposures (parental control and nurturance) for each of four binary outcomes (TV viewing and personal computer [PC] use on weekdays and weekend days). Given that it is not possible to determine whether TV viewing and PC use occurred independently or concurrently, we performed all analyses for the two outcomes separately. It should be noted that, because Cerin and MacKinnon^29^ have argued that mediation can still occur in the absence of an association between the exposure variable and the outcome variable, we continued with the full mediation models, even in the absence of a direct association. Mediation was assumed to have occurred if a previously significant association between X and Y is no longer significant. Complete mediation of the pathway between the exposure and the outcome occurs when the effect of X on Y controlling for M (path c’) becomes zero.

Models were adjusted for child BMI *z*-score, household IMD, and parent SV. Confidence intervals (CIs) were based on robust standard errors, which took account of the clustering of participants within schools. Mediation statistics, including the proportion mediated and the indirect effect, were obtained using a modified version of the user-written Stata “binary_mediation” command, and bias-adjusted 95% CIs were derived using bootstrapping methods.^30^ Preliminary analyses showed that including either parent or child sex had very little effect on the overall results or on *R*^2^ in the mediation models, and there was no evidence of an interaction with either child or parent sex. Therefore, all analyses are presented for the entire sample. Analyses were performed in Stata 12.0 (Statacorp LP, College Station, TX).

## Results

A total of 954 parents (708 mothers and 246 fathers) provided sufficient information to be included in the analyses (Table 1). Mothers had higher scores on the nurturance scale, compared to fathers (32.2 vs. 30.5; *p*<0.001). There was evidence to suggest that parents included in the analyses were more controlling (31.1 vs. 31.8; *p*=0.001), felt less able to limit SV (13.3 vs. 13.6; *p*=0.0149), and were from households with higher IMD scores (15.4 vs. 14.3; *p*<0.001; Supplementary Table 1) (see online supplementary material at http:www.liebertpub.com).

**Table 1.** Characteristics of and Number of SV Devices in the Homes of Participants by Gender

	Mean	SD	Mean	SD	Difference in means	95% CI	*p* value^*^
Adults	Male parent (*n*=246)		Female parent (*n*=708)				
Age, years	39.6	6.1	37.3	5.5	2.3	1.48 to 3.14	**<0.001**
BMI	26.4	4.3	25.2	4.6	1.2	0.52 to 1.86	**<0.001**
IMD score^a^	13.1	11.3	14.7	12.4	−1.6	−3.37 to 0.14	0.072
Parental self-efficacy to limit SV	13.6	1.6	13.7	1.7	−0.10	−0.35 to 0.15	0.427
Parental control	3.7	1.0	3.6	1.1	0.10	−0.05 to 0.26	0.190
Parental nurturance	30.5	4.3	32.2	3.7	−1.73	−2.29 to −1.17	**<0.001**
Media equipment (all)	13.4	6.0	13.1	5.1	0.31	−0.47 to 1.09	0.436
TVs	4.7	2.4	4.9	2.3	−0.17	−0.51 to 0.17	0.331
PCs (including laptops and tablets)	2.4	1.5	2.2	1.4	0.20	−0.01 to 0.40	0.060
Games consoles	2.2	1.7	2.3	1.7	−0.09	−0.34 to 0.16	0.474
Music players	2.5	1.9	2.4	1.7	0.15	−0.10 to 0.39	0.236
Smart phones	1.6	1.0	1.4	0.9	0.20	0.06 to 0.34	**0.004**

Children	Boys (*n*=493)		Girls (*n*=461)		Difference in means	95% CI	*p* value^*^
Age, years	6.0	0.4	6.0	0.4	0.02	−0.03 to 0.08	0.458
BMI-z score	0.25	1.0	0.23	0.9	0.02	−0.1.0 to 0.14	0.705
IMD score^a^	14.5	12.4	14.2	11.8	0.32	−1.22 to 1.86	0.688

^a^ A high score shows greater levels of social deprivation.

^*^ *p* value from *t*-tests.

IMD, index of multiple deprivation; SV, screen viewing; TVs, televisions; PCs, personal computers; SD, standard deviation; CI, confidence interval.

SV was similar in boys and girls on weekdays and at the weekend (Table 2). On weekdays, 90% of children met guidelines of less than 2 hours of TV per day. At weekends, however, this figure decreased to 55% for boys and 58% for girls. Computer use by girls and boys was similar, with approximately 75% of both genders reportedly having some exposure to PCs at home on weekend days, compared with 61% on weekdays.

**Table 2.** Demographic Data of Screen Viewing among Parents and Children

	Weekday												
	Male						Female						
	<2 hours		2 hours or more				<2 hours		2 hours or more				
	*n*	%	*n*	%			*n*	%	*n*	%			*p* value^*^
Parent TV	185	75.2	61	24.8			494	69.8	214	30.2			0.105
Child TV	439	89.1	54	10.9			421	91.3	40	8.7			0.238

	≤30 minutes		31 minutes to 2 hours		>2 hours		≤30 minutes		31 minutess to 2 hours		>2 hours		
	*n*	%	*n*	%	*n*	%	*n*	%	*n*	%	*n*	%	
Parent PC	79	32.1	91	37.0	76	30.9	283	40.0	281	39.7	144	20.3	**0.002**

	Not used		Any use				Not used		Any use				
	*n*	%	*n*	%			*n*	%	*n*	%			
Child PC	183	37.1	310	62.9			183	39.7	278	60.3			0.413

	Weekend day												
	Male						Female						
	<2 hours		2 hours or more				<2 hours		2 hours or more				
	*n*	%	*n*	%			*n*	%	*n*	%			
Parent TV	119	48.4	127	51.6			346	48.9	362	51.1			0.893
Child TV	271	55.0	222	45.0			268	58.1	193	41.9			0.324

	≤30 minutes		31 minutes to 2 hours		>2 hours		≤30 minutes		31 minutes to 2 hours		>2 hours		
	*n*	%	*n*	%	*n*	%	*n*	%	*n*	%	*n*	%	
Parent PC	17	6.9	102	41.5	127	51.6	46	6.5	300	42.4	362	51.1	0.955

	Not used		Any use				Not used		Any use				
	*n*	%	*n*	%			*n*	%	*n*	%			
Child PC	120	24.3	373	75.7			117	25.4	344	74.6			0.711

^*^ *p* value from chi-square tests showing differences in associations between males and females.

TV, television; PC, personal computer.

Logistic regression analysis indicated that for parental TV watching behavior on weekdays, each unit increase in parental control score was associated with a 26% reduction in the odds of a child watching >2 hours of TV per weekday (odds ratio [OR], 0.74; 95% CI, 0.58–0.93; Table 3). Parental efficacy to influence SV was independently associated with the odds of children watching <2 hours of TV per weekday (OR, 0.75; 95% CI, 0.68–0.83), and there was some evidence that parental efficacy mediated the path between parental control and children's weekday TV viewing behavior. Including the potential mediator reduced the odds of a child watching >2 hours of TV per weekday to 20% (OR, 0.80; 95% CI, 0.64–1.00). The proportion of the total effect mediated was 23%. There was limited evidence that parental nurturance was associated with children's TV watching (OR, 0.95; 95% CI, 0.90–1.02). There was, however, some evidence that the path between these variables was mediated by parental efficacy to influence SV (mediated OR, 0.99; 95% CI, 0.93–1.05; proportion of the total effect mediated was 71%; Table 3).

**Table 3.** Logistic Regression Predicting Child TV Watching Behavior on Weekdays by Parental Control and Parental Nurturance, with Parental Efficacy To Restrict Screen Viewing as a Potential Mediator

Parental control	Adjusted^a^ (with clustering)		
Step 1: Outcome=child TV on weekdays^b^	OR	95% CI	*p*
Parental control (C)	0.74	0.58–0.93	0.009
	Pseudo-R^2^, 0.118; *p*<0.001		
Step 2a: Predictor: parental control	Coeff	95% CI	*p*
(a) Outcome: efficacy to influence screen viewing (A1)	0.25	0.14–0.36	<0.001
	*R*^2^, 0.033; *p*<0.001		
Step 2b: Mediator on outcome	OR	95% CI	*p*
Efficacy to influence screen viewing (B)	0.75	0.68–0.83	<0.001
	Pseudo-*R*^2^, 0.143; *p*<0.001		
Step 3: Outcome=child TV on weekdays^b^	OR	95% CI	*p*
Parental control (C’)	0.80	0.64–1.00	0.056
Efficacy to influence screen viewing	0.77	0.69–0.85	<0.001
	*R*^2^, 0.150; *p*<0.001		
Mediation statistics:		Bias-corrected 95% CI	
Indirect effect	−0.04	−0.06 to −0.02	
Proportion of total effect mediated	0.23		

Parental nurturance	Adjusted^a^ (with clustering)		
Step 1: Outcome=child TV on weekdays^b^	OR	95% CI	*p*
Parental control (C)	0.95	0.90–1.02	0.139
	Pseudo-*R*^2^, 0.109; *p*<0.001		
Step 2a: Predictor: parental nurturance	Coeff	95% CI	*p*
(a) Outcome: efficacy to influence screen viewing (A1)	0.12	0.09–0.15	<0.001
	*R*^2^, 0.085; *p*<0.001		
Step 2b: Mediator on outcome	OR	95% CI	*p*
Efficacy to influence screen viewing (B)	0.75	0.68–0.83	<0.001
	Pseudo-*R*^2^, 0.143; *p*<0.001		
Step 3: Outcome=child TV on weekdays^b^	OR	95%CI	*p*
Parental nurturance (C’)	0.99	0.93–1.05	0.672
Efficacy to influence screen viewing	0.76	0.68–0.84	<0.001
	*R*^2^, 0.143; *p*<0.001		
Mediation statistics:		Bias-corrected 95% CI	
Indirect effect	−0.07	−0.10 to −0.04	
Proportion of total effect mediated	0.71		

^a^ Adjusted for child BMI *z*-score, IMD, and parental weekday TV viewing.

^b^ >2 hours versus 2 hours or less.

TV, television; IMD, index of multiple deprivation; OR, odds ratio; Coeff, coefficient; CI, confidence interval.

Children's weekday PC use was not predicted by parental control (OR, 0.99; 95% CI, 0.89–1.10), and there was no evidence of mediation by parental efficacy to influence SV (indirect effect, −0.02; 95% CI, −0.04 to −0.01; proportion of total effect mediated=2.89%; Table 4).

**Table 4.** Logistic Regression Predicting Child PC Use on Weekdays by Parental Control and Parental Nurturance, with Parental Efficacy To Restrict Screen Viewing as a Potential Mediator

Parental control	Adjusted^a^ (with clustering)		
Step 1: Outcome=child PC use on weekdays^b^	OR	95% CI	*p*
Parental control (C)	0.99	0.89–1.10	0.864
	Pseudo-*R*^2^, 0.023; *p*<0.001		
Step 2a) Predictor: parental control	Coeff	95% CI	*p*
(a) Outcome: Efficacy to influence screen viewing (A1)	0.25	0.14–0.36	**<0.001**
	*R*^2^, 0.031; *p*<0.001		
Step 2b: Mediator on outcome	OR	95% CI	*p*
Efficacy to influence screen viewing (B)	0.88	0.82–0.95	**0.001**
	Pseudo-*R*^2^ 0.031; *p*<0.001		
Step 3: Outcome=child PC use on weekdays^b^	OR	95% CI	*p*
Parental control (C’)	1.02	0.91–1.14	0.709
Efficacy to influence screen viewing	0.88	0.81–0.81	**0.001**
	*R*^2^, 0.031; *p*<0.001		
Mediation statistics:		Bias-corrected 95% CI	
Indirect effect	−0.02	−0.04 to −0.01	
Proportion of total effect mediated	2.89		

Parental nurturance	Adjusted^a^ (with clustering)		
Step 1: Outcome=child PC use on weekdays^b^	OR	95% CI	*p*
Parental nurturance (C)	0.97	0.94–1.00	0.044
	Pseudo-R^2^, 0.026; *p*<0.001		
Step 2a: Predictor: parental nurturance	Coeff	95% CI	*p*
(a) Outcome: efficacy to influence screen viewing (A1)	0.12	0.09–0.15	**<0.001**
	*R*^2^, 0.084; *p*<0.001		
Step 2b: Mediator on outcome	OR	95% CI	*p*
Efficacy to influence screen viewing (B)	0.88	0.82–0.95	**0.001**
	Pseudo-*R*^2^, 0.031; *p*<0.001		
Step 3: Outcome=child PC use on weekdays^b^	OR	95% CI	*p*
Parental nurturance (C’)	0.98	0.95–1.02	0.268
Efficacy to influence screen viewing	0.89	0.82–0.96	**0.004**
	*R*^2^, 0.032; *p*<0.001		
Mediation statistics:		Bias-corrected 95% CI	
Indirect effect	−0.03	−0.05 to −0.01	
Proportion of total effect mediated	0.41		

^a^ Adjusted for child BMI *z*-score, IMD, and parental weekday PC use.

^b^ Some use versus no use.

PC, personal computer; IMD, index of multiple deprivation; OR, odds ratio; Coeff, coefficient; CI, confidence interval.

Each unit increase on the parental nurturance scale was associated with a 3% decrease in the odds of a child using a PC on weekdays (OR, 0.97; 95% CI, 0.94–1.00). There was evidence that parental efficacy to influence SV partially mediated the path between these two variables, with a significant proportion (41%) of the total effect being mediated (revised OR, 0.98; 95% CI, 0.95–1.02; Table 4).

Neither parental control nor nurturance were directly associated with children's weekend TV viewing, although there was evidence that the direct pathway in both models was mediated by parental efficacy to influence SV (Supplementary Table 2) (see online supplementary material at http:www.liebertpub.com). Weekend PC use was not significantly predicted by either of the parenting variables, nor was there any evidence of mediation by parental efficacy to influence SV (Supplementary Table 3) (see online supplementary material at http:www.liebertpub.com).

## Discussion

In this study, 45% of boys and 42% of girls spent more than 2 hours watching TV on a weekend day, with just over half of parents exceeding this threshold. There was strong evidence that parental control was associated with child weekday TV viewing, with each unit increase on the parent control scale associated with a 26% reduction in the odds that a child spent >2 hours per day watching TV. Parental self-efficacy to limit SV was associated with 5- to 6-year-old children's weekday and weekend TV watching and PC use on weekdays, with each unit increase on the scale being associated with a 25% reduction in the odds that a child spent >2 hours watching TV on weekdays. At weekends, the equivalent reduction was 12%. Parental self-efficacy mediated the path between both control (23%) and nurturance (71%) and weekday TV viewing. For PC use, there was some evidence that parental nurturance was associated with child PC use, but the association was weak. There was no evidence of an association between parental control and PC use on either weekdays or weekend days. Parental self-efficacy to limit SV was strongly associated with PC time, with each unit on the scale associated with a 12% reduction in the odds that children were using PCs. Self-efficacy mediated 41% of the path between nurturance and weekday PC use. Overall, the results highlight weak effects for the more distal control and nurturance and an important role for the proximal parental self-efficacy variable. It is important to note that parental self-efficacy may vary for different behaviors and in different situations. Strategies to boost self-efficacy for specific behaviors may help to change behaviors and enhance overall behavior change, even when the parent is under stress.

Our results suggest that developing an intervention focusing on increasing parents' awareness of the importance of limiting SV and enhancing confidence to say “no” or to offer their children alternative activities to SV that may reduce SV, which may also aid in the prevention of obesity. The impact of higher parental self-efficacy was greater on a weekday (25% reduction in odds of watching more than 2 hours of TV) than a weekend day (12% reduction). This difference may reflect the greater time available for children to SV at the weekend because, even if a parent were to assert their efficacy to offer valued alternatives on some occasions to SV, there are likely to be other times in the weekend day when their child is permitted to engage in SV behavior. Conversely, on weekdays, a parent may only need to assert their efficacy once or twice to set up alternative activities (a club or playing outside), which can fill a few hours before dinner and reduce SV for that day. The findings presented here therefore complement emerging research showing that parenting programs concentrating on parental skills to manage screen time among older children^31^ are useful and suggest that programs that focus on both weekday and weekend SV could be helpful.

These data raise several methodological issues in relation to the measurement of parenting styles. First, our preliminary analysis indicated that the control measure had low internal consistency. The measure was developed in the United States,^26^ and the low internal consistency may reflect differences between UK and US respondents, suggesting that further UK measurement work is needed. Second, the derivation and interpretation of categories of parenting styles may warrant reconsideration. Previous research has shown that authoritative parenting, which is characterized by higher levels of control and nurturance, is associated with positive child outcomes.^12,13,32–35^ The data presented in this article suggest that the variables typically used to derive parenting styles do not seem to impact on SV behaviors in the same way that they do on other behaviors. The lack of association seems inconsistent with findings from previous parenting studies, but might be a consequence of using the disaggregated variables. Previous studies have forced the derivation of four groups based on median splits of control and parenting variables. The median split approach also makes it hard to identify how control and nurturance are independently associated with health behaviors, and future studies should consider reporting how nurturance and control are independently associated with health outcomes. The results of this study are therefore broadly consistent with the recent recommendations of Power and colleagues for the development of new measures to measure parenting constructs in a variety of different contexts.^36^ Finally, our results appear to broadly support the model proposed by Sleddens and colleagues^22^ that any impact of parenting styles on health outcomes is likely to be mediated by parenting practices.

### Strengths and Limitations

The major strength of this study is the availability of SV time for both parent and child along with parental perceptions of factors considered to be important in the role of limiting the amount of children's SV behavior. This facilitated the examination of such factors as they relate to children who have recently begun primary (elementary) school. The study is limited by the parental report of child SV, which is likely to be subject to an under-reporting bias. We have attempted to minimize this by including the parental SV in the models, given that it is reasonable to assume that a parent who tends to underestimate their own SV time is likely to underestimate their child's by a similar amount. The cross-sectional nature of the study design limits the ability to identify the direction of associations. Finally, it is important to recognize that there are several limitations of applying mediation analyses to cross-sectional data with binary outcomes. Based on the recommendations for Cerin and Makinnon,^29^ we applied the product-of-coefficients method to estimate any mediation effects, but are aware of the possible limitations of this approach.^37,38^ While recognizing the limitations of mediation analysis, we are confident that the overall results for the model used are robust.

## Conclusions

Results presented here show that parental self-efficacy to limit SV was associated with lower levels of SV among 5- to 6-year-old children, and that parental self-efficacy partially mediated the association between parental control and TV viewing. Results suggest that the development of strategies to increase parental self-efficacy to limit screen time may be useful for reducing screen time and preventing the development of behaviors that increase the risk of childhood obesity.

## Supplementary Material

Supplemental data

Supplemental data

Supplemental data
