# Supplementary material for: Parental Control, Nurturance, Self-Efficacy, and Screen Viewing among 5- to 6-Year-Old Children: A Cross-Sectional Mediation Analysis To Inform Potential Behavior Change Strategies
Source: Child Obes. 2015 Apr 1;11(2):139–47. doi: 10.1089/chi.2014.0110 (PMC4382711; doi:10.1089/chi.2014.0110)
Supplement: Supplemental data [file Supp_Table1.pdf]

Supplementary Data

Supplementary Table 1. Characteristics of and Number of Screen Viewing Devices in the Homes of Participants Included in Analysis versus Participants Excluded

	Mean	SD	Mean	SD	Difference in means	95% CI	*p* value[†]
**Adults**	**Included**		**Excluded**				
Age (years)	37.9	5.7	36.5	5.6	−1.38	−2.53 to −0.23	0.02
BMI	25.5	4.6	24.6	4.3	−0.86	−1.73 to 0.01	0.05
IMD score[a]	14.3	12.1	17.9	15.4	3.62	1.74 to 5.51	**<0.001**
Parental self-efficacy to limit SV	13.6	1.7	13.3	2.0	−0.42	−0.75 to −0.08	**0.01**
Parental control	3.7	1.1	3.1	1.1	−0.53	−0.85 to −0.21	**0.001**
Parental nurturance	31.8	3.9	31.1	4.7	−0.66	−1.46 to 0.14	0.10
Media equipment (all)	13.1	5.3	12.4	5.7	−0.72	−1.74 to 0.30	0.17
TVs	4.8	2.4	5.1	2.6	0.26	−0.19 to 0.72	0.26
PCs (including laptops and tablets)	2.3	1.4	1.9	1.3	−0.42	−0.69 to −0.16	**0.002**
Games consoles	2.3	1.7	2.3	1.7	0.02	−0.30 to 0.35	0.89
Music players	2.4	1.7	2.0	1.5	−0.33	−0.65 to −0.01	**0.04**
Smart phones	1.4	1.0	1.2	1.0	−0.18	−0.36 to 0.09	**0.06**
**Children**	**Included**		**Excluded**		**Difference in means**	**95% CI**	***p* value***
Age (years)	6.0	0.43	6.0	0.23	0.015	−0.03 to 0.06	0.55
BMI z-score	0.24	0.93	0.34	0.10	0.100	−0.02 to 0.22	0.10

[a]A high score shows greater levels of social deprivation.

*p value from t-tests.

IMD, index of multiple deprivation; SV, screen viewing; TVs, televisions; PCs, personal computers; SD, standard deviation; CI, confidence interval.
