# Supplementary material for: Parental Control, Nurturance, Self-Efficacy, and Screen Viewing among 5- to 6-Year-Old Children: A Cross-Sectional Mediation Analysis To Inform Potential Behavior Change Strategies
Source: Child Obes. 2015 Apr 1;11(2):139–47. doi: 10.1089/chi.2014.0110 (PMC4382711; doi:10.1089/chi.2014.0110)
Supplement: Supplemental data [file Supp_Table2.pdf]

Supplementary Table 2. Logistic Regression Predicting Child TV Watching Behavior on Weekend Days by Parental Control and Parental Nurturance, with Parental Efficacy To Restrict Screen Viewing as a Potential Mediator

**Parental control**	**Adjusted[a] (with clustering)**		
Step 1: Outcome = child TV on weekend days[b]	OR	95% CI	*p*
Parental control (C)	0.90	0.79–1.01	0.083
	Pseudo-R^2, 0.110; $p < 0.001$		
Step 2a: Predictor: parental control	Coeff	95% CI	*p*
a) Outcome: Efficacy to influence screen viewing (A1)	0.25	0.14–0.36	**<0.001**
	R^2, 0.032; $p < 0.001$		
Step 2b: Mediator on outcome	OR	95% CI	*p*
Efficacy to influence screen viewing (B)	0.88	0.82–0.95	**0.001**
	Pseudo-R^2, 0.115; $p < 0.001$		
Step 3: Outcome = child TV on weekend days[b]	OR	95% CI	*p*
Parental control (C')	0.92	0.81–1.04	0.194
Efficacy to influence screen viewing	0.89	0.82–0.96	**0.002**
	R^2, 0.116; $p < 0.001$		
Mediation statistics:		Bias-corrected 95% CI	
Indirect effect	−0.02	−0.04 to −0.01	
Proportion of total effect mediated	0.26		
**Parental nurturance**	**Adjusted[a] (with clustering)**		
Step 1: Outcome = Child TV on weekend days[b]	OR	95% CI	*p*
Parental nurturance (C)	0.99	0.96–1.03	0.720
	Pseudo-R^2 0.108; $p < 0.001$		
Step 2a: Predictor: parental nurturance	Coeff	95% CI	*p*
(a) Outcome: efficacy to influence screen viewing (A1)	0.12	0.09–0.15	**<0.001**
	R^2, 0.083; $p < 0.001$		
Step 2b: Mediator on outcome	OR	95% CI	*p*
Efficacy to influence screen viewing (B)	0.88	0.82–0.95	**0.001**
	Pseudo-R^2, 0.115; $p < 0.001$		
Step 3: Outcome = child TV on weekend days[b]	OR	95% CI	*p*
Parental nurturance (C')	1.01	0.97–1.05	0.665
Efficacy to influence screen viewing	0.88	0.81–0.95	**0.002**
	R^2, 0.115; $p < 0.001$		
Mediation statistics:		Bias-corrected 95% CI	
Indirect effect	−0.03	−0.05 to −0.01	
Proportion of total effect mediated	2.34		

[a]Adjusted for child BMI z-score, IMD, and parental weekend TV viewing.

[b]>2 hours versus 2 hours or less.

TV, television; IMD, index of multiple deprivation; OR, odds ratio; Coeff, coefficient; CI, confidence interval.
