# Supplementary material for: Parental Control, Nurturance, Self-Efficacy, and Screen Viewing among 5- to 6-Year-Old Children: A Cross-Sectional Mediation Analysis To Inform Potential Behavior Change Strategies
Source: Child Obes. 2015 Apr 1;11(2):139–47. doi: 10.1089/chi.2014.0110 (PMC4382711; doi:10.1089/chi.2014.0110)
Supplement: Supplemental data [file Supp_Table3.pdf]

Supplementary Table 3. Logistic Regression Predicting Child PC Use on Weekend Days by Parental Control and Parental Nurturance, with Parental Efficacy To Restrict Screen Viewing as a Potential Mediator

**Parental control**	**Adjusted[a] (with clustering)**		
Step 1: Outcome = child PC use on weekend days[b]	OR	95% CI	*p*
Parental control (C)	1.04	0.95–1.15	0.329
	Pseudo-R^2, 0.003; $p = 0.3776$		
Step 2a: Predictor: parental control	Coeff	95% CI	*p*
(a) Outcome: Efficacy to influence screen viewing (A1)	0.25	0.14–0.36	**<0.001**
	R^2 0.035; $p < 0.001$		
Step 2b: Mediator on outcome	OR	95% CI	*p*
Efficacy to influence screen viewing (B)	0.95	0.87–1.03	0.208
	Pseudo R^2, 0.004; $p = 0.2576$		
Step 3: Outcome = child PC use on weekend days[b]	OR	95% CI	*p*
Parental control (C')	1.06	0.97–1.17	0.206
Efficacy to influence screen viewing	0.94	0.87–1.03	0.165
	R^2, 0.05; $p = 0.2724$		
Mediation statistics:		Bias-corrected 95% CI	
Indirect effect	−0.01	−0.02 to 0.002	
Proportion of total effect mediated	−0.32		
**Parental nurturance**	**Adjusted[a] (with clustering)**		
Step 1: Outcome = child PC use on weekend days[b]	OR	95% CI	*p*
Parental nurturance (C)	0.99	0.95–1.03	0.721
	Pseudo-R^2, 0.031; $p = 0.4179$		
Step 2a: Predictor: parental nurturance	Coeff	95%CI	*p*
(a) Outcome: efficacy to influence screen viewing (A)	0.12	0.09–0.15	**<0.001**
	R^2, 0.089; $p < 0.001$		
Step 2b: Mediator on outcome	OR	95% CI	*p*
Efficacy to influence screen viewing (B)	0.94	0.87–1.03	**0.208**
	Pseudo-R^2, 0.004; $p = 0.2576$		
Step 3: Outcome = child PC use on weekend days[b]	OR	95% CI	*p*
Parental nurturance (C')	1.00	0.96–1.04	0.967
Efficacy to influence screen viewing	0.95	0.87–1.04	**0.242**
	R^2, 0.043; $p = 0.3713$		
Mediation statistics:		Bias-corrected 95% CI	
Indirect effect	−0.01	−0.04 to 0.006	
Proportion of total effect mediated	0.88		

[a]Adjusted for child BMI z-score, IMD, and parental weekend PC use.

[b]Some use versus no use.

PC, personal computer; IMD, index of multiple deprivation; OR, odds ratio; Coeff, coefficient; CI, confidence interval.
